# Understanding Rejection between First-and-Second-Grade Elementary Students through Reasons Expressed by Rejecters

**DOI:** 10.3389/fpsyg.2017.00462

**Published:** 2017-04-04

**Authors:** Francisco J. García Bacete, Virginia E. Carrero Planes, Ghislaine Marande Perrin, Gonzalo Musitu Ochoa

**Affiliations:** ^1^Department of Developmental, Educational and Social Psychology and MethodologyUniversitat Jaume I, Castellón de la Plana, Spain; ^2^GREI Interuniversity Research Group, Universitat Jaume ICastellón de la Plana, Spain; ^3^Department of Education and Social Psychology, Pablo de Olavide UniversitSevilla, Spain

**Keywords:** reasons for peer rejection, grounded theory, norms, group, preferences, identity, unfamiliarity, early elementary education

## Abstract

**Objective:** The aim of this research was to obtain the views of young children regarding their reasons for rejecting a peer.

**Method:** To achieve this goal, we conducted a qualitative study in the context of theory building research using an analysis methodology based on Grounded Theory. The collected information was extracted through semi-structured individual interviews from a sample of 853 children aged 6 from 13 urban public schools in Spain.

**Results:** The children provided 3,009 rejection nominations and 2,934 reasons for disliking the rejected peers. Seven reason categories emerged from the analysis. Four categories refer to behaviors of the rejected children that have a cost for individual peers or peer group such as: direct aggression, disturbance of wellbeing, problematic social and school behaviors and dominance behaviors. A further two categories refer to the identities arising from the preferences and choices of rejected and rejecter children and their peers: personal identity expressed through preferences and disliking, and social identity expressed through outgroup prejudices. The “no-behavior or no-choice” reasons were covered by one category, unfamiliarity. In addition, three context categories were found indicating the participants (interpersonal–group), the impact (low–high), and the subjectivity (subjective–objective) of the reason.

**Conclusion:** This study provides researchers and practitioners with a comprehensive taxonomy of reasons for rejection that contributes to enrich the theoretical knowledge and improve interventions for preventing and reducing peer rejection.

## Introduction

Maintaining a minimum number of meaningful, positive and lasting interpersonal relationships (Baumeister and Leary, 1995), and belonging to groups (Nesdale, 2007) are persistent motivations for people. During early elementary school children make new relationships and start being actively involved in various peer networks (Ladd, 2005). However, whereas some children are accepted and included in different groups, other children are rejected and excluded by their peers (Gifford-Smith and Brownell, 2003; Abrams et al., 2005; Asher and McDonald, 2009; Killen et al., 2009). Peer rejection is a common peer experience that predicts maladjustment outcomes, mood disorders and victimization in childhood and adulthood (Leary, 2001; McDougall et al., 2001; Saarento et al., 2013). Knowing what drives some children to reject/dislike others is therefore a question of interest (Mikami et al., 2010).

This study aims to understand the reasons given by first-and-second-grade children for rejecting some of their peers. Most studies using peer assessment, attributional approach or social exclusion have been descriptive of children aged 8 or older, as younger children’s assessments of their classmates have been considered little reliable (Nesdale and Duffy, 2011). In contrast, we think that, instead of asking the reasons why one thinks that others are rejected or oneself is rejected, which is the information collected by the aforementioned techniques, it is more suitable to ask the rejecters themselves the reasons why they dislike some peers. Selman (2003), in his interpersonal coordination theory, stated that most children aged 6–8 years are cognitively aware of their own thoughts, motives and feelings in social interactions. Besides, the sociometric methods have shown to be useful to assess the “attractions” and “repulsions” between children as young as three (Cillessen, 2009). We used open-ended questions to collect the reasons given by the rejecters. Then, in order to analyze these reasons we chose a qualitative theory building approach to seize the whole richness of the responses, avoiding pre-established categories. Working with 6-and-7-year old children will provide valuable information to implement specific actions designed to prevent and reduce peer rejection at early ages (García Bacete et al., 2014).

McDougall et al. (2001, p. 214) referred to peer rejection as “dislike on the part of one’s peers which may or may not be accompanied by varying degree of victimization, exclusion, or intentional isolation from peer activities.” This definition includes the three characteristics of peer rejection: (1) Rejection is based on dislikes, which are attitudes and not necessarily behaviors (Leary, 2005). In this study we use indistinctly dislike and rejection. (2) Rejection occurs between peers who know each other in the context of a group, which indicates a common history (Doosje et al., 2002). The often private character of disliking requires focusing on who rejects, and the fact that disliking occurs between known peers requires conducting the study in the ecological settings where the peer interactions take place, like classrooms. (3) When rejection is perpetrated by a significant number of peers, it is conceptualized as peer rejection.

However, for many years the predominant conceptualization in rejection research has been based on the deficits of the rejected child that contribute to her/his social difficulties (Asher, 1990; Bierman, 2004). Thus, most studies have focused on the rejected person, largely by examining the correlates and consequences of rejection (Coie et al., 1990; Bierman, 2004), and other questions such as perceptions of being rejected (Guerra et al., 2004), reactions to rejection (Sandstrom and Zakriski, 2004), or problematic social situations for peer-rejected students (Martín-Antón et al., 2016). When attention is given to the rejecter, the focus has generally been on explicit rejection behaviors (Asher et al., 2001; Lev-Wiesel et al., 2013). It is therefore unsurprising that research into the reasons for rejection has also focused on the rejected person and on behavioral correlates of rejection as the causes of rejection (Rubin et al., 2006). In contrast, few studies directly asked rejecters to give their reasons for rejecting known peers in real situations (Monjas et al., 2008).

### Correlates of Rejection and Other Reasons for Rejection

For many years scholars have been keen to learn about which behavioral characteristics lead children and adolescents to be accepted or rejected by their peers (Asher and McDonald, 2009). This is what is known as the correlates of rejection (Coie et al., 1990; Bierman, 2004; Rubin et al., 2006). Newcomb et al. (1993) found that high levels of aggression and withdrawal and low levels of sociability and cognitive abilities are associated with rejected peer status. Bierman (2004) summarized this set of reasons as she stated that rejected children can be more argumentative, disruptive, and aggressive, more socially awkward and insensitive, less skillful in engaging in prosocial play, and/or have more negative interactions with teachers than their peers. In general, the rejected person’s characteristics and behaviors are considered to invite rejection from others (Asher, 1990; Newcomb et al., 1993; McDougall et al., 2001; Mikami et al., 2010). However, correlates cannot be interpreted so readily as causes (McDougall et al., 2001), and can frequently be better understood as consequences of the rejection itself (Orue and Calvete, 2011). Chang (2004) found that some rejected children are popular and central members of their group, which suggests that rejection is a function of those who reject rather than of the rejected person’s behaviors. Thus, rejection is not a property of the rejected child or a characteristic of her/his behavior (Dirks et al., 2007).

Alongside, other studies presented children with hypothetical situations or experimental designs and asked them else to select or rate causes of social failure or peer rejection, as did social attribution studies (Elig and Frieze, 1975; Goetz and Dweck, 1980; Earn and Sobol, 1984), or to reason the legitimacy of social exclusion behaviors in manipulated hypothetical situations, as did studies on social exclusion based in the social-cognitive domains (Abrams et al., 2005; Smetana, 2006; Killen, 2007; Abrams and Rutland, 2008; Killen et al., 2009). In the attributional studies, children’s responses are usually interpreted in accordance with the three dimensions proposed by Weiner, locus, stability, and control (e.g., “She played better,” external, unstable, controllable). But above all the attributional approach emphasizes the success/failure of the rejected child’s behavior in a specific social situation (e.g., managing to “play with,” “get on well with”), whereas peer rejection cannot be interpreted in terms of the failure neither of the rejected child, because “not being liked” does not depend on the child her/himself but on the other, nor of the rejecter because nobody can accept everyone (Leary, 2001). In the social exclusion studies, the reasons for exclusion are classified according to moral reasons such as treating the ingroup and the outgroup members in the same fair and equitable way, social-conventional reasons such as identification with the group and the way it functions, and personal reasons such as preferences and attributions of intentions to others (Horn, 2006; Killen et al., 2009). These contributions are valuable; however, the focus of these studies is the behavior of rejection or exclusion, and not the rejection as an attitude. In addition, in this type of studies, the reasons are derived from interpretation of situations and evaluation of behaviors (Turiel, 1998; Smetana, 2006). Both approaches use strongly structured designs in which the children are asked to explain why a certain event and behavior occur and the presented situations and reasons may have little meaning for the children. Moreover, the analysis of children’s responses is based on pre-established dimensions or categories.

### Evaluation by the Rejecters and Group Context

So far, little is known about the reasons that may explain the process of rejection as a result of an interpersonal interaction in a social group, as proposed by Bierman (2004). This perspective implies two relevant issues: First, research on the reasons for rejection must focus on the rejecter child; and second, the social context in which peer interactions occur influences rejection.

The first question is related to the principle of relational evaluation put forward by Leary (2001, 2005). According to this principle, any form of rejection, regardless of the behaviors or traits of the rejecters or rejected child, is a state of relatively low relational evaluation in which a person does not consider his/her relationship with another individual as valuable. Many of our decisions to reject reflect personal preferences based on our attitudes, interests, abilities, goals, and previous social experiences (Leary, 2001; Nucci, 2001; Scandroglio et al., 2008). Similarly, Asher and McDonald (2009) stated that the basis for peer rejection is not the behavior of the rejected child but rather the others’ interpretation that the relationship with that child does not properly satisfy their needs (e.g., “Does this child seek to have influence in ways that are not acceptable for me?”).

As for the second issue, it is important to recognize that neither the rejecter nor the rejected student are on their own, but they are members of groups (class group, class subgroups, social categorization groups as gender, birth place, etc…) (Bourdieu, 1985; Nesdale, 2011). Several authors have concluded that a complete model of peer rejection could be obtained only through the understanding of social context influences (García Bacete et al., 2014; Mulvey et al., 2014). Killen et al. (2013) suggested that what seems to be interpersonal rejection might actually reflect group rejection.

Mikami et al. (2010) described the processes by which groups influence peer rejection: cognitive biases held by the accepted peer group, deviation from peer group norms; and social dominance hierarchy in the peer group. Abrams and Rutland (2008) found that individuals who do not fit well into their own group are highly likely to be rejected. Poor adaptation to a group can occur either because the person does not maintain typical relationships with her peers (e.g., “being shy,” see Rubin et al., 2006), or because she contributes little or nothing, or harms the group (e.g., “violate the natural tendency to cooperate within the group,” see Levine and Moreland, 1994). In turn, Nesdale (2007, 2011) found that belonging to other groups entailed greater probability of rejection, either due to preference for one’s own group, or due to prejudice toward other groups (gender, ethnic group etc.) (Brown and Bigler, 2005; Scandroglio et al., 2008; Mulvey et al., 2010).

In summary neither the rejection nor the reasons for rejection can be understood as a characteristic of a person or of a behavior, or as an attribution or cause, or as an evaluation of behaviors, social situations, or social failures. Peer rejection is an attitude or a feeling, a negative or low relational evaluation, and the reasons are an attempt to explain this relational evaluation in the group context. We agree with Asher and McDonald (2009, p. 235) in that “scholars who study the behavioral correlates of acceptance and rejection rarely discuss the ways that the behaviors they study are powerful because they speak to peoples’ fundamental needs,… that it might suggest other characteristics that are relevant to acceptance and rejection but have not yet been studied.”

### Open–Ended Question and Qualitative Method

It is therefore important to include open–ended questions to study reasons for rejection since, as pointed out by Elig and Frieze (1979), they are more meaningful and allow for greater spontaneity than the aforementioned approaches, and they do not hint at any reasons that children have not even thought about.

This strategy has been used in a few studies. Smith (1950), a teacher, asked her fourth-grade class open-ended questions about the reasons for their positive and negative sociometric choices. The children’s responses showed that verbal abusiveness, rule violations, and bullying were associated with negative nominations. Monjas et al. (2008), using the responses of fifth-and-sixth-grade children to the questions “Which of your classmates do you not like and why,” developed a taxonomy of 15 reasons for peer rejection ranging from unfriendliness and lack of companionship to aggressive, dominating and antisocial behaviors. Feinberg et al. (1958) asked sixth-and-seventh-grade students to describe the classmates with whom they felt uncomfortable or annoyed, found that the traits fighting, disruptive, conceited, and silly were associated with negative status. These studies represented a contribution since they analyzed the reasons in situations where rejected children and rejecters interacted on a daily basis, which were highly meaningful and very different from the hypothetical situations and anonymous protagonists with no background history nor future exposed in the aforementioned more structured methods (Doosje et al., 2002). However, the reasons for rejection in these studies are still presented in a descriptive way without going deeper into the interconnections between categories, they are largely interpreted in terms of the behavior of the rejected child without taking into consideration the rejecter and rejected children’s group context.

A qualitative approach is needed to understand the reasons that prompt children to reject or dislike some peers. Grounded Methodology developed by Glaser and Strauss (1967), has proved capable of generating basic conceptual categories from data that explain the processes that occur in complex social situations (Carrero et al., 2012). From this perspective neither a predetermined taxonomy of possible reasons nor an explanatory framework to interpret them is necessary. Instead of that, the Grounded Theory starts from the data provided by the social participants and, by applying the constant comparison of the incidents found (the reasons given by the rejecters), makes emerge substantive categories which are compared to new incidents. These successive comparisons continue until achieving the theoretical saturation of the data given by the participants. This process yields an underlying structure that explains the variability of the elicited reasons. Data collection, coding process, integration of categories, and construction of theory are thus guided by methodology as it emerges.

This study aims to understand the reasons given by first-and-second-grade children for rejecting some of their peers. In order to analyze these reasons the conducted study focuses on: (1) Defining peer rejection as an attitude or a feeling, a negative or low relational evaluation. (2) Asking the rejecters themselves the reasons why they dislike some peers. (3) Including the influence of social context in which peer interactions occur. (4) Applying a qualitative theory building approach to seize the whole richness of the responses and avoid pre-established categories.

## Materials and Methods

### Design and Participants

In this study we used a theory building qualitative approach with a methodology of analysis based on Grounded Theory (Glaser and Strauss, 1967) consisting of analyzing the information provided by a sample of children through the codification, comparison, and conceptualization of data coming from their speech to form categories and establish relationships between them (Strauss and Corbin, 2007). The sample has been selected by applying *master selection criteria* (age, sociometric type) and *heterogeneity-homogeneity criteria* (sex, education level, place of residence, and socioeconomic status) according to the objectives of the research (Valles, 2000; Suárez et al., 2013, 2016) (**Table 1**). An incidental sampling was used to select public elementary schools situated in urban districts with average socioeconomic level close to the four universities where the researchers were conducting a broader study that included a large intervention later on. These criteria and the extend of the sample was designed taking into account the need to ensure the *point of redundancy* (Lincoln and Guba, 1985) where the new information analyzed is redundant with the previous data and could be integrated into the existing categories.

**Table 1:** Description of the sample of respondents (*n* = 853) according to place of residence, education level, age, gender, sociometric type, number of classrooms, number of schools, and socioeconomic status.

Cities		Valencia *n^a^* = 244 (28.6)	Palma *n* = 272 (31.9)	Sevilla *n* = 243 (28.5)	Valladolid *n* = 94 (11.0)	Total *n* = 853
Education level	First	244 (100)	147 (54.0)	107 (44.0)	94 (100)	592 (69.4)


	Second	–	125 (46.0)	136 (56.0)	–	261 (30.6)


Age	5	22 (9.0)	16 (5.9)	12 (4.9)	6 (6.4)	56 (9.5)


	6	222 (91.0)	233 (85.7)	215 (88.5)	88 (93.6)	497 (84.0)


	7	–	23 (8.5)	16 (6.6)	–	39 (6.5)


Gender	Boy	129 (52.9)	131 (48.2)	120 (49.4)	50 (53.2)	430 (50.4)


	Girl	115 (47.1)	141 (51.8	123 (50.4)	44 (46.8)	423 (49.6)


Sociometric Type	Average	172 (70.5)	164 (60.3)	180 (74.1)	73 (77.7)	589 (69.0)


	Preferred	29 (11.9)	28 (10.3)	20 (8.2)	8 (8.5)	85 (10.0)


	Rejected	29 (11.9)	31 (11.4)	31 (12.8)	11 (11.7)	102 (12.0)


	Neglected	6 (2.5)	38 (14.2)	6 (2.5)	1 (1.1)	51 (6.0)


	Controversial	8 (3.3)	11 (4.0)	6 (2.5)	1 (1.1)	26 (3.0)


School SES^b^		M2	M2	M2	M2	M2

^a^*n = sample (%). ^b^SES = Socioeconomic status: M1 = Medium-low; M2 = Medium; M3 = Medium-high*.

Finally, to guarantee the validity of the results and the consistency of the emergent categories several procedures, based on the recommendations claimed by Glaser (1978, 1992) and Thomas (2006), were applied in this study: first, to find a way to avoid bias in the researcher during the process of coding without losing his theoretical sensitivity and, second, to verify the consistency of the final categories. To achieve the first goal, the *substantive coding* phase (Strauss and Corbin, 2007) was developed by a researcher who was trained previously in coding technique but who was not an expert in peer rejection (third author) while for the *selective* and *theoretical coding* phases, three experts in peer rejection and qualitative methodology joined the analysis work (first, second, and fourth author). Secondly, to verify the consistency of the final categories we applied a *coding consistency check* (Valles, 2000; Thomas, 2006) by consulting two experts in rejection and bullying and then calculated the interrater agreement (Dubé, 2008) using Cohen’s kappa statistic (κ) in accordance with the guidelines proposed by Landis and Koch (1977).

Participants were 939 pupils of both sexes, aged 5–7 years, who were studying in 40 first-and-second-grade classrooms of 13 schools in four Spanish cities: Valencia, Palma de Mallorca, Sevilla and Valladolid.

Of the 939 initial subjects 86 pupils (9.2%) did not respond. Forty-six of them because they did not have the parental permission, were not at school during the assessment, or left the school during the study (although they continued to be nominated by their classmates). The other 40 children gave nominations of acceptation but not of rejection. The final sample of respondents consisted of 853 pupils (49.6% girls) (*M*_age_ = 6.76 years, *SD* = 0.67).

### Data Collection

In each city, two trained evaluators (research collaborators graduate in educational psychology) gathered the data by means of 20-min individual interviews in which they asked two questions extracted from the sociometric questionnaire (García Bacete and González, 2010): (1) Who in your class do you like least? (2) Why don’t you like (classmate’s name)? To answer the first question, the child identified all the classmates he or she did not like on the class photographs. After that, the researcher asked the student the second question for each of the classmates nominated negatively and wrote down verbatim the child’s reasons. The information gathered in the interviews allowed compiling a list of reasons verbalized by the pupils to explain the rejection toward their peers. To ensure that all students provide a comparative number of reasons, only a maximum of five reasons were used per student.

Each school left a private room at disposal for carrying out the interviews. The present study was conducted in accordance with the 1964 Helsinki declaration and its later amendments or comparable ethical standards, with the approval of the management board of schools, the educational inspection services, the Department of Education of the Regional Government of Valencia (Spain), the Childhood Observatory of the Regional Government of Andalusia (Spain), the Socio-Educational Institute Foundation s’Estel of the Government of the Balearic Islands (Spain); and the Observatory School Coexistence of the Autonomous Government of Castilla y León (Spain). Review and approval from an ethics committee was not required as per the institutional and national requirements. Participation in the study was voluntary. All subjects gave written informed consent. The required authorizations from the education authorities, the schools, and the children’s families were obtained.

### Analytic Strategy

First, the list of reasons given by the participants was read in detail by the researchers to familiarize themselves with the content and to approach a first understanding of the “themes” and details of the subject. After that, in the first step of the analysis we used the *substantive coding*. The substantive codes break down (fracture the data) and then conceptually grouping it into codes that then become the theory which explains what is happening in the data (Glaser, 1978). During substantive coding the data are examined, and compared for similarities and differences. The researcher compares incident to incident with the purpose of establishing the underlying uniformity and its varying conditions (Glaser, 1978). In our study we grouping under the same label the reasons exposed by the children that use common key words used by the participants (e.g., friend, “She doesn’t want to be my friend,” “He is Rob’s friend”), or that had a certain similarity (e.g., lack of hygiene, “He’s always got a runny nose”; “she doesn’t clean her teeth”). This process yielded 10 groups of reasons or categories (**Table 2**).

**Table 2:** Initial grouping of reasons for rejection.

–	**NO FRIENDSHIP**: She isn’t my friend; he doesn’t want to be my friend; he’s Rob’s friend;…


–	**NO RELATIONSHIP**: I don’t talk to her much; he never sits next to me; when I come she goes away; he doesn’t come any more; we don’t play; she plays with other children;…


–	**I DON’T LIKE…**: I don’t like her at all; I don’t like being with her much; I don’t get on with her; there’s something about him I don’t like; I don’t like the way he runs;…


–	**PERSONAL CHARACTERISTICS**: She’s really bad tempered; he’s mad; he gets angry; she spends all day crying; he gets anxious; he acts cocky; she never keeps her promises; she isn’t friendly; he isn’t much fun; he’s really tedious; she’s boring; she doesn’t leave me in peace; he doesn’t share; she’s bossy; she thinks she’s the boss; he tells lies;…


–	**PHYSICAL CHARACTERISTICS/PHYSICAL APPEARANCE**: She’s a gypsy; he’s got horrible teeth; he’s a boy; she’s got big ears; I don’t like her hair; he walks funny; I don’t like his face; she wears glasses; she wears horrible clothes;…


–	**LACK OF HYGIENE**: He’s always got a runny nose; he’s really dirty; she doesn’t clean her teeth;…


–	**POOR ACADEMIC SKILLS**: she doesn’t do her homework; he doesn’t understand anything; he’s thick; she copies; he reads really badly; she doesn’t know much about school stuff;…


–	**BAD BEHAVIOR/DISRUPTIVE CONDUCT**: She takes my things; she breaks my friends’ things; the teacher always gets cross with him; he gets told off; she spits; he throws sand; he’s very naughty; she’s bad; he’s very rough; she threatens us; she cheats; she insults; he shouts; he makes fun of me; she doesn’t shut up in class; he swears; he talks nonsense;…


–	**PHYSICAL AGGRESSION**: She pinches; he fights; he pushes us; she pulls our hair; he throws juice at us; he hits;…


–	**OTHERS**: I don’t know; I don’t remember;…

Applying the constant comparison method (Glaser, 1978; Carrero et al., 2012), we then created subgroups within the same category. Depending on the frequency and distinctive meaning of the reasons that appeared under the same label, they form a subcategory within the existent category or a new independent category. After this first categorization the resulting categories still needed to be refined, therefore we continue applying the constant comparative technique to the data (**Table 3**).

**Table 3:** Serotypes and phylogenetic tree of select swine STEC strains analyzed using the FDA-ECID array.

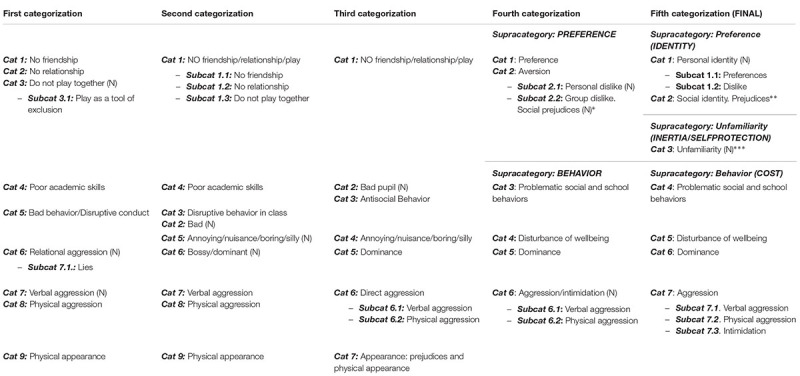

*The original order of the categories in the categorizations First, Second, and Third has been changed to make visible the parallelism with the FINAL categorization. *N* = New; ^∗^it includes Cat 7 of Third categorization; ^∗∗^it coincides with Cat 7 of Third categorization; ^∗∗∗^it comes from Cat 1 of Fourth categorization*.

As the reader can see on **Table 3**, during this continuous process of open coding, the configuration and definition of the list of categories suffered constant changes. For example, in the second categorization, the *bad behavior/disruptive conduct* category divided into two categories: Bad (“stupid,” “He’s a cheat,” “She steals”) and *disruptive behavior in class* (“He doesn’t keep quiet in classroom,” “He gets punished”). In the third categorization, these two new categories combined with the category *poor academic skills* and gave way to two categories: *bad pupil* and *antisocial behavior*.

With each new category configuration or change to labels or definitions, we revised each reason one by one, and the number of unclassified reasons gradually disappeared. By this point (third categorization), practically all the main reasons were present: *aggression, dominance, annoyance, antisocial behavior, bad pupil, no friendship/relationship/play, appearance*.

At this point of the analysis, relationships between concepts were analyzed, identifying the position of the actors (rejecter or rejected child) as regard their disliking or liking referring to friendship, relationship or play, and defining the links between categories and subcategories hierarchically, establishing a series of main categories or supracategories (*selective coding*). In this way, we first distinguished between preference and aversion and then gradually differentiated two principal sets of reasons for rejection: *behavior* (supracategory of rejection because of what the rejected child does, says, or tries to do), *preference* (supracategory of rejection because of what the rejected child s/he likes).

The last step in the analysis consisted in validating the final theoretical scheme, confirming the relationships between categories, removing data and filling in categories that still needed further refinement and development (*theoretical coding*). For example, from the *preference* category we decided to remove reasons that expressed little or no contact between rejecter and rejected child because above all they revealed an absence of preference (choice) or of shared activity (behavior), for which they could not be in the *preference* nor in the *behavior* supracategories, thus forming a third independent supracategory, *unfamiliarity*. Simultaneously it became clear that *preference* is a positive choice and *dislike* is a negative choice and that both would underlie personal identity, whereas prejudices against people belonging to or representing certain social categories would account for the base of social identity, which led to the categories *personal identity* and *social identity*.

In this theoretical phase another important aspect had gained relevance. It is observed that all the reasons exposed contain information concerning the children who participate (“She hits me,” “He hits us”), the different times or intensity of the event (“Sometimes she does not play with me,” “He never plays with me”), and the different levels of subjectivity-objectivity of what is said (“I don’t like him,” “She didn’t invite me to her birthday party”). From these observations, three context categories emerged: *participants*, *impact*, and *subjectivity–objectivity*. The context categories can be represented as transversal axes to each reason category.

Finally, to calculate the interrater agreement we sent the definition of each category to the two expert raters and asked them to assign a list of 500 reasons randomly to the final set of categories.

## Results

The pupils provided 3,009 rejection nominations, of which 2.5% contained no reasons for rejection, or the respondent claimed not to know the reasons for the rejection. The analysis of the remaining 2,934 reasons produced seven reason categories, arranged in three supracategories, and three context categories. The interrater agreement reached with the two expert raters was strong (κ = 0.90 with rater 1, and κ = 0.81 with rater 2).

### Reason Supracategories and Categories

#### Supracategory: Behavior (Cost) (74.1%)

The reasons of this category express rejection of the other because of what s/he does, says, or tries to do and these behaviors represent threats to or attacks on social and school expectations and norms (“She takes things away,” “He’s bad at reading”), personal and group wellbeing (“He bothers”, She speaks when we’re working”), autonomy (“He bosses people about,” “She pushes me around”), and physical and emotional safety (“He hits,” “She makes fun of everybody”). Only when these behaviors are interpreted as costs for the personal and groups norms and functioning, or do not contribute to satisfy the individual and group needs, the child becomes the object of rejection.

##### Problematic social and school behaviors category (17.4%)

This category highlights simultaneously antinormative and abnormal social and school behaviors of the rejected child. On one hand, such behaviors go against what is considered appropriate and desirable, against general or specific social and school expectations and norms, which reflect the breach of social and school rules norms (“She steals things,” “She makes the teacher angry”). On the other hand, they refer to deficits in the social and school skills necessary for relational and educational success or a deficiency in fulfilling expectations and achieving goals (“He doesn’t leave me anything,” “He reads really badly”).

##### Disturbance of wellbeing category (18.4%)

This category refers to the rejected child’s behaviors that make people feel uncomfortable and angry and obstruct people to achieve their objectives (“He says silly things,” “She is always interrupting”). The reasons in this category are characterized by frequent behaviors of low intensity that interfere with what one wants, when and how one wants, and in the end cause personal or group discomfort.

##### Dominance category (4.5%)

Dominance is understood as behaviors of the rejected child that aim to impose what is to be done, influence others for one’s own advantage or strengthen one’s own ego at the expense of others (“He bosses people about,” “He acts cocky with me”).

##### Aggressive category (33.8%)

This category is defined by direct behaviors of the rejected child that cause personal or physical harm, or insecurity. They may be verbal and gestural aggressive aimed to humiliate others or damage their reputation (4.7%) (“He insults,” “She shouts at me”), physical aggression aimed to cause physical damage (23.1%) (“He hits,” “He spits”), or intimidation aimed to frighten the person through threats or abuse (6.0%) (“She treats me badly,” “He threatens”). All these behaviors share the purpose of harming, but in the case of intimidation, it is the fear of what may occur that makes feel the harm beforehand.

#### Supracategory: Preference (Identity) (18.6%)

This supracategory refers to what the rejecter or the rejected child likes and is. It consists of two categories, *personal identity* and *social identity*, which are based on two different attraction processes: personal attraction, which derives from the idiosyncratic preferences arising in interpersonal relationships; and social attraction, which derives from the degree to which an individual represents the prototype of his or her group.

In this supracategory the reason are firstly situated in the affective frame of likings/dislikes of the rejecter or of the rejected child (in fact, the rejecter’s perception of the rejected child’s preferences). In a second moment, the preferences become cognitive or/and behavioral choices, which can involve direct rejection (“I don’t want to be his friend”) or indirect (“I play with my friends”). Finally, when the preferences and choices are systematically used and based on exclusion norms the personal identity becomes reinforced. Moreover, when these systematical preferences are based on negative reputations and social prejudices, then the social identity becomes strengthened. This identity process may lead to situations where differences strongly clash, people forget about ethics and egalitarian treatment, and rejection settles down. Here too the influence of the peer context or the group is present.

##### Personal identity category (14.5%)

In this category the subject shows that her/his likings are different from those of the people s/he rejects. This category has two subcategories: preference and dislike.

Preference subcategory (3.7%). This subcategory expresses the rejecter’s likings, enjoyments or choices (I like it/him/her) or those of the rejected child (s/he likes it/him/her) referring to a person, relationship, friendship, activity or play, that extends to the group (we like it/him/her, I like them, they like me, s/he likes us). In the reasons of this category, the rejecter affirms her/his preferences or those of others (“I like playing football,” “He plays with his friends”).

Dislike subcategory (10.8%). This category expresses the rejecter’s volition (I don’t want/like) or the rejected child’s volition as perceived by the rejecter (s/he doesn’t want/like) not to establish a relationship or share friendship and play. This intention can also be extended to their groups (we don’t want, they don’t want). In this subcategory the rejecter shows her/his own dislikes or those of his/her group, or reacts to the dislikes of other or others (“I don’t like their games,” “She doesn’t let me play,” “He doesn’t want to play with me”).

##### Social identity category (4.1%)

In this category the dislike of other children is based on their belonging to a social group or category (“She’s a girl”) or on their doing activities typical of those same groups (“He plays girls’ games”), in the absence of other more specific reasons. These reasons actually express stereotypes and prejudices against those who are not like me or us, or belong to other group (“She’s Romanian,” “He’s new”).

#### Supracategory: Unfamiliarity (Inertia/Self-protection) (7.4%)

This supracategory is a set of reasons that express little or absence of choices-sharing and of activities-sharing (We aren’t…, we don’t go…, we don’t play…), for which they cannot be in the *preference* nor can they be in the *behavior* supracategories. This supracategory is formed by a unique category, unfamiliarity. It includes reasons that reflect low interest in little known others, or hesitation to make new relationships, which are manifested in not searching for contacts and shared activities (“I don’t know him/her,” “We don’t play together”).

**FIGURE 1:**
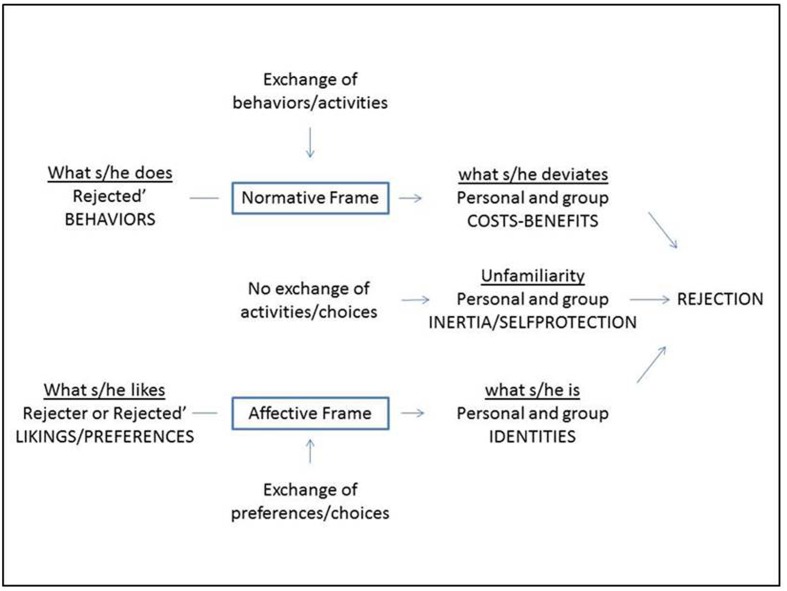
**Sources and paths of motivation for peer rejection**.

**Figure 1** describes the final map of categories where three paths to the motivation for peer rejection can be observed: first path to rejection lies in the rejected children’s deviant behavior (What s/he does, says, tries) in the context of their personal and group relationships (costs of the behavior); second path to rejection is built on the preferences and choices of the rejecters and rejected children, defining what they are (identities). The third path to rejection consists of the absence of behavioral interactions and choices (unfamiliarity).

### Context Categories

The context categories are referred to conditions in which rejection occurs. These categories modify as well as increase the diversity of reasons given by the rejecters. The context must be considered to deeply understand the reasons given by the interviewed children about why they reject or dislike some peers.

The *participants* context distinguishes between interpersonal rejection and group rejection. The interpersonal rejection occurs when the participants are the rejecter–rejected pair (33.7%) (“He hits me”) or trios in which in the rejection between rejecter and rejected a third pupil is included (4.1%) (“She hits my friend”). The group rejection occurs when the rejecter or the rejected child or both are in a group. The groups can be number-limited or formed by known peers (5.7%) (“He hits my friends”), or be larger collectives, or relate to general statements (56.5%) (“He hits everybody”).

The *impact* context includes frequency and intensity of the event and distinguishes between low, medium and high impact. Low impact (8.6%) refers to single or occasional cases (“He pushed me once”), or low intensity (“He’s a little bad”). Medium impact occurs when there are no intensity or frequency indicators, or is expressed in indefinite or third person (66.1%) (“She doesn’t let others play”). High impact refers to maximum frequency or intensity (25.2%) (“He doesn’t lend things to anybody”).

The third context category relates to the *subjectivity–objectivity* feature of the reason, whether the meaning of the reason is a function of the subject who rejects or a function of the event or object for which rejection occurs. We use the term subjectivity when the rejecter’s feeling and thinking are part of the reason and the rejecter acts as a judge or interpreter. In this way, the valence of the reason can be different (positive, negative, or neutral), depending on the evaluator. Subjective reasons may be: total when referring to the whole rejected person (8.7%) (“I don’t like her”) or partial when referring to only one aspect (21.9%) (“He’s annoying”). On the contrary, other reasons focus on the rejected child as something external, and the rejecter only describes the reason as a narrator or observer of an event in which the rejected child participated. The descriptive reasons can be: general and imprecise (45.8%) (“She calls names”) or specific or precise (23.7%) (“She plays with Danny”).

## Discussion

From the above said several contributions of this study can be deduced. First of all, it major part of reasons refers to what the rejected does, in line with the studies of the correlates of rejection and the social attribution (Earn and Sobol, 1984; Coie et al., 1990; Bierman, 2004). However, in contrast to those studies, rejection here does not appear to be the direct result of what the rejected does, but of the relational evaluation of this behavior done by the rejecters (Leary, 2001), of how they interpret that this behavior affects their needs and the group functioning (Levine and Moreland, 1994; Asher and McDonald, 2009), and its degree of typicality in comparison to the behavior of the own group or of other groups (Nesdale, 2007; Abrams and Rutland, 2008). This path of rejection highlights the power of the behavioral interactions and the deviation from the norms to provoke peer rejection, since the classmates interpret them as costs to the interpersonal relationships and the group functioning (Levine and Moreland, 1994; Ladd, 2005). The information provided by the context categories strengthens the utility of using the rejecter and the group approaches in the study of peer rejection.

Secondly, the rejecters provided two types of reasons that do not usually appear in the traditional studies on rejection (e.g., Bierman, 2004), -namely the reasons for rejection due to *preference* and to *unfamiliarity*-, because the classic studies focus mainly on the rejected children’s behavior (what they do, say or try to do). The second path highlights the power of the preferences both in the personal domain and in the social categorization to provoke peer rejection (Smetana, 2006; Nesdale, 2007; Abrams and Rutland, 2008; Mikami et al., 2010), since the children interpret them as likings and choices that crystallize in personal and group identities (Scandroglio et al., 2008; Nesdale, 2011). The third path highlights that the social inertia toward choosing and doing what has already been preferred and done (Bourdieu, 1985), or the fear and mistrust to what is unknown or unfamiliar (Gifford-Smith and Brownell, 2003), may also lead to rejection (Allport, 1954). The reasons included in those two supracategories are external to the rejected children’s behavior since they focus on the rejecters’ attractions and choices, (or those that the rejecters attribute to the rejected), and on what does not befall (not sharing activities, not making choices). These new reasons could appear owing to having put the interest on the rejecter, and because rejecters and rejected children are known peers, members of the same classroom. The rejecters’ answers “I don’t know” or “no response” are other examples of reasons external to the rejected child’s behavior.

Thirdly, the fact that children at this age, in their explaining their reasons for peer rejection, turn to arguments simultaneously referring to the self, the group, and stereotypes, confirm the results of the theory of social-cognitive domain to explain the exclusion (Turiel, 1998; Smetana, 2006; Killen, 2007), and strengthen the idea that the social judgments are not a characteristic of one stage but emerge simultaneously in development, unlike to Kohlberg’s (1984) model. In fact, the reasons for peer rejection show a parallelism with the three social-cognitive domains: The reasons based on *personal identity* match with personal domain (Nucci, 2001; Gifford-Smith and Brownell, 2003), the reasons based on the rejected child’s *behavior* with the social normative domain (Abrams et al., 2005; Ladd, 2005), the reasons based on *social identity* with the moral domain (Brown and Bigler, 2005; Mulvey et al., 2010; Hatfield and Rapson, 2011), and the reasons based on *unfamiliarity* with both personal and social domains.

Fourthly, the content and the form of the reasons reflect the three characteristics of peer rejection: private evaluation, group influence, context of known peers.

*Private evaluation*: Rejection is above all a private and attitudinal evaluation (Leary, 2001, 2005). The presence of reasons that make it difficult for the observer to identify certain situations as rejection puts in evidence this private character. This is the case of reasons under the heading *do not know/no response*; of reasons expressed in past tense that refer to a memory and not a present reality; and of reasons included in the *unfamiliarity* category. The private evaluative nature is also present in the reasons where the rejecter’s opinion is an inseparable part of the content, where the rejection is not so much due to the behavior or the event but rather to the interpretation the rejecter makes of it, since the same behavior (“he’s always singing”) may be evaluated as positive, negative, or neutral by different evaluators. In other reasons, like those in the *social identity* category, the content itself of the reason includes the evaluation, in this case discriminatory or prejudiced. Thus, the rejecter’s perspective becomes indispensable to know the reasons for peer rejection, because since rejection is essentially a private evaluation, the reasons are arguments for rejection only if the rejecter feels and thinks like it.

*Group influence*: Although the methodology used for the data collecting had an interpersonal basis, the analysis methodology allowed the emergence of clear indicators of the influence of the others and of the group in the reasons, showing that reasons can also be based on the group and/or norms (Horn, 2006). The influence of the group in the interpersonal system mentioned by Mikami et al. (2010) is observed in the transversal and majority presence in all the categories of group reasons, reasons in which groups act as spokespersons or recipients (62.2% of the *participants context*). Even though interpersonal and group reasons overlap in all categories and permit tracking this group basis (Killen et al., 2013), some categories reflect the group influence with more clarity, as in the cases of the *problematic social and school behaviors* category that reports on ingroup dynamics (Abrams and Rutland, 2008) and the *social identity* category on outgoup dynamics (Nesdale, 2007, 2011).

*Context of known peers*: Finally, the fact that peer rejection occurs between known peers who share identity or history is corroborated by the constant references in the reasons to particular classmates or relatives, terms such as ‘friends’ or ‘us,’ and known situations and norms. This condition of known peers in peer rejection has two implications. Precisely the fact that the children share a classroom and will probably continue to do so for some time makes rejection a highly socially significant situation, strongly stable and with negative outcomes (McDougall et al., 2001; García Bacete et al., 2014). Simultaneously this condition requires the reasons for peer rejection to be studied in their context (García Bacete et al., 2014; Mulvey et al., 2014).

### Limitations and Future Directions

Some limitations of this study would refer to the studied populations, other to the use of data collecting techniques and other to group influence. Differential analyses need to be performed on the reason and context categories according to gender (Sureda et al., 2009), broadening the study to other ages (8–10, 11–13, and older), examining the differences in the reasons for rejection between minority and majority groups (e.g., children from minority/majority ethnic group, with/without special educational needs, rejected/average).

As seen above, many of the reasons are expressed briefly and imprecisely, with no type of indicator. It may be possible that children do not need more precise or detailed arguments; however, it would be interesting to carry out in-depth interviews to allow the children to explain what they do not like about their classmates in a broader and more precise way. It may be a potentially useful method for finding out whether they think the rejected child knows s/he is not liked, whether they do or say anything to make him or her know that they dislike him/her, whether children have criteria that they use consistently when thinking about how they feel about another child, and other similar questions.

Finally, we need to progress in the study of the influence of the classroom and the others. As discussed, our study has provided indicators, but it remains far from systematically undertaking this goal. Two alternatives: first, through the realization of multilevel studies in which being a member of a classroom represents the higher level, this study will help to know whether the motivational structure of rejection in a given classroom differs from the one in another classroom. Secondly, by examining if there is consensus across children on what they say about a particular child.

## Conclusion

The present study reinforces that rejection is part of children’s daily life. Only 4.5% of the participants did not name negatively any children. Moreover, 94% of the rejecters express reasons for rejection. The richness of reasons for rejection as well as the subsequent comprehensive taxonomy could have been obtained only through the conceptual and methodological decisions adopted in this study, namely: understanding rejection as a relational evaluation, focusing on the rejecters as informants, studying rejection in its ecological context, and using qualitative methodology in the data collection and analysis.

In brief, main contributions of this study are: (a) Peer rejection is external to the rejected child; that is, what the rejected child does or says does not lead directly or inevitably to rejection. (b) Rejection occurs during the exchange of activities/behaviors and preferences/choices in a group context, or even in the absence of exchange, between a rejecter or group of rejecters and a rejected child or group of rejected children. (c) The sources and pathways that lead to rejection are the rejecters’ interpretations of these exchanges in terms of interpersonal and group costs, negative personal and social identities, personal or group inertia or self-protection, evidencing a great interdependence between the interpersonal and the group levels. (d) Through the study of the reasons we could observe that peer rejection is a heterogeneous social reality, in the number of participants and the link between them, the frequency or intensity of it, and the degree of objectivity/subjectivity with which the rejecter refers to the exchanges. (e) The development of children at age 6 already displays a rich knowledge of the socio-cognitive domains used to explain the peer relationships, so that our findings revealed personal reasons, normative or socio-conventional reasons and moral reasons.

In summary, peer rejection at this age can be understood as a negative relational evaluation, expressed by individual or group rejecters, toward individually or group rejected children, in the form of a specific or general description, or a partial or total judgment, both unidirectionally and bidirectionally, and with a variable impact. Such evaluation operates simultaneously in the personal, social and moral domains (Mulvey et al., 2014), as an exercise of personal autonomy (Nucci, 2001), or in response to attacks or threats (Ladd, 2005), or as a prejudicial aversion (Mulvey et al., 2010), or even in the absence of interactions and arguments (Williams and Zadro, 2001). In definitive, the study provides researchers and practitioners with a comprehensive taxonomy of reasons for rejection that contributes to the theoretical construct of peer rejection and the design of interventions for preventing and reducing peer rejection.

## Author Contributions

FG: Led and designed the study, coordinated data collection, performed the analysis and interpretation of the data, and drafted the manuscript. VC: Revised the analytic strategies, participated in the analysis and interpretation of the data, and helped to draft the manuscript. GMD: Participated in data collection, participated in the analysis and interpretation of the data, and helped to draft the manuscript. GMO: Contributed to the analysis and interpretation of the data, revised critically the study, and participated in drafting the manuscript. All authors approved the final manuscript as submitted.

## Conflict of Interest Statement

The authors declare that the research was conducted in the absence of any commercial or financial relationships that could be construed as a potential conflict of interest.

## References

[B1] Abrams D. Hogg M. A. Marques J. M. (2005). “A social psychological framework for understanding social inclusion and exclusion,” in *The Social Psychology of Inclusion and Exclusion*, eds Abrams D. Marques J. M. Hogg M. A. (Philadelphia, PA: Psychology Press), 1–26.

[B2] Abrams D. Rutland A. (2008). “The development of subjective group dynamics,” in *Intergroup Relations and Attitudes in Childhood through Adulthood*, eds Levy S. R. Killen M. (Oxford: Oxford University Press), 47–65.

[B3] Allport G. W. (1954). *The Nature of Prejudice.* Reading, MA: Addison–Wesley.

[B4] Asher S. R. (1990). “Recent advances in the study of peer rejection,” in *Peer Rejection in Childhood*, eds Asher S. R. Coie J. D. (Cambridge: Cambridge University Press), 3–14.

[B5] Asher S. R. McDonald K. L. (2009). “The behavioral basis of acceptance, rejection, and perceived popularity,” in *Handbook of Peer Interactions, Relationships, and Groups: Social, Emotional, and Personality Development in Context*, eds Rubin K. H. Bukowski W. M. Laursen B. (New York, NY: Guilford) 232–249.

[B6] Asher S. R. Rose A. J. Gabriel S. W. (2001). “Peer rejection in everyday life,” in *Interpersonal Rejection*, ed. Leary M. (New York, NY: Oxford University Press), 105–142.

[B7] Baumeister R. F. Leary M. R. (1995). The need to belong: desire for interpersonal attachments as a fundamental human motivation. *Psychol. Bull.* 117 497–529. 10.1037/0033-2909.117.3.497 7777651

[B8] Bierman K. L. (2004). *Peer Rejection: Developmental Processes and Intervention Strategies.* New York, NY: The Guilford Press.

[B9] Bourdieu P. (1985). The social space and the genesis of groups. *Theory Soc.* 14 723–744. 10.1007/bf00174048

[B10] Brown C. S. Bigler R. S. (2005). Children’s perceptions of discrimination: a developmental model. *Child Dev.* 76 533–553. 10.1111/j.1467-8624.2005.00862.x 15892777

[B11] Carrero V. E. Soriano R. Trinidad A. (2012). *Teoría Fundamentada “Grounded Theory”: El Desarrollo de la Teoría desde la Generalización Conceptual [Grounded Theory”: The Construction of Theory from Conceptual Generalization].* Madrid: Centro de Investigación Sociológica, 37.

[B12] Chang L. (2004). The role of classroom norms in contextualizing the relations of children’s social behaviors to peer acceptance. *Dev. Psychol.* 40 691–702. 10.1037/0012-1649.40.5.691 15355159

[B13] Cillessen A. H. N. (2009). “Sociometric methods,” in *Handbook of Peer Interactions, Relationships, and Groups*, eds Rubin K. H. Bukowski W. M. Laursen B. (New York, NY: Guilford), 82–99.

[B14] Coie J. D. Dodge K. A. Kupersmidt J. (1990). “Peer group behavior and social status,” in *Peer Rejection in Childhood*, eds Asher S. R. Coie J. D. (Cambridge: Cambridge University Press), 17–58.

[B15] Dirks M. A. Treat T. A. Weersing R. (2007). Integrating theoretical, measurement, and intervention models of youth social competence. *Clin. Psychol. Rev.* 27 327–347. 10.1016/j.cpr.2006.11.002 17270330

[B16] Doosje B. Spears R. Ellemers N. (2002). Social identity as both cause and effect: the development of group identification in response to anticipated and actual changes in the intergroup status hierarchy. *Br. J. Soc. Psychol.* 41 57–76. 10.1348/014466602165054 11970774

[B17] Dubé J. E. (2008). Evaluación del acuerdo interjueces en investigación clínica. Breve introducción a la confiabilidad interjueces [Evaluation of interrater reliability in clinical research. A brief introduction to interrater reliability]. *Rev. Argent. Clin. Psicol.* 17 75–80.

[B18] Earn B. M. Sobol M. P. (1984). A categorical analysis of children’s attributions for social experience. *Psychol. Rec.* 40 173–186.

[B19] Elig T. W. Frieze I. H. (1975). *A Multi-Dimensional Scheme for Coding and Interpreting Perceived Causality for Success and Failure Events/Coding Scheme of Perceived Causality, CSPC.* Washington, DC: American Psychological Association.

[B20] Elig T. W. Frieze I. H. (1979). Measuring causal attributions for success and failure. *J. Pers. Soc. Psychol.* 37 621–634. 10.1037/0022-3514.37.4.621

[B21] Feinberg M. R. Smith M. Schmidt R. (1958). An analysis of expressions used by adolescents of varying economic levels to describe accepted and rejected peers. *J. Genet. Psychol.* 93 133–148. 10.1080/00221325.1958.10532411 13575793

[B22] García Bacete F. J. González J. (2010). *Evaluación de la Competencia Social Entre Iguales [Assessment of the Peer Social Competence: the Sociometry and Other Measures].* Madrid: TEA Ediciones.

[B23] García Bacete F. J. Marande Perrin G. Sanchiz Ruiz M. L. Sureda García I. Ferrá Coll P. Jiménez Lagares I. (2014). *El Rechazo Entre Iguales en su Contexto Interpersonal: Una Investigación con Niños y Niñas de Primer Ciclo de Educación Primaria [Peer Rejection in its Interpersonal Context: A Research Work on Children in First and Second Grades of Elementary Education].* Castellón: Fundación Dávalos–Fletcher.

[B24] Gifford-Smith M. E. Brownell C. A. (2003). Childhood peer relationships: social acceptance, friendships, and peer networks. *J. Sch. Psychol.* 41 235–284. 10.1016/S0022-4405(03)00048-7

[B25] Glaser B. G. (1978). *Theoretical Sensitivity: Advances in the Methodology of Grounded Theory.* Mill Valley, CA: Sociology Press.

[B26] Glaser B. G. (1992). *Basic of Grounded Theory Analysis: Emergence vs. Forcing.* Mill Valley, CA: Sociology Press.

[B27] Glaser B. G. Strauss A. L. (1967). *The Discovery of Grounded Theory.* Aldine de Gruyter, CA: Sociology Press.

[B28] Goetz T. E. Dweck C. (1980). Learned helplessness in social situations. *J. Pers. Soc. Psychol.* 39 246–255. 10.1037/0022-3514.39.2.246 7411393

[B29] Guerra V. S. Asher S. R. DeRosier M. E. (2004). Effect of children’s perceived rejection on physical aggression. *J. Abnorm. Child Psychol.* 32 551–563. 10.1023/B:JACP.0000037783.88097.69 15500033

[B30] Hatfield E. Rapson R. L. (2011). “Equity theory in close relationships,” in *Handbook of Theories of Social Psychology*, eds Lange P. A. M. van Kruglanski A. W. Higgins E. T. (London: Glyph International), 200–217.

[B31] Horn S. (2006). Group status, group bias, and adolescents’ reasoning about treatment of others in school contexts. *Int. J. Behav. Dev.* 30 208–218. 10.1177/0165025406066721

[B32] Killen M. (2007). Children’s social and moral reasoning about exclusion. *Curr. Dir. Psychol. Sci.* 16 32–36. 10.1111/j.1467-8721.2007.00470.x

[B33] Killen M. Mulvey K. L. Hitti A. (2013). Social exclusion: a developmental intergroup perspective. *Child Dev.* 84 772–790. 10.1111/cdev.12012 23170901

[B34] Killen M. Rutland A. Jampol N. S. (2009). “Social exclusion in childhood and adolescence,” in *Handbook of Peer Interactions, Relationship, and Groups*, eds Rubin K. H. Bukowski W. M. Laursen B. (New York, NY: Guilford Press) 249–266.

[B35] Kohlberg L. (1984). *Essays on Moral Development: The Psychology of Moral Development–The Nature and Validity of Moral Stages*, Vol. Vol. 2. San Francisco: Harper and Row.

[B36] Ladd G. W. (2005). *Children’s Peer Relations and Social Competence.* New Haven, CT: Yale University Press.

[B37] Landis J. R. Koch G. G. (1977). The measurement of observer agreement for categorical data. *Biometrics* 33 159–174. 10.2307/2529310 843571

[B38] Leary M. R. (2001). “Toward a conceptualization of interpersonal rejection,” in *Interpersonal Rejection*, ed. Leary M. R. (New York, NY: Oxford University Press), 3–20.

[B39] Leary M. R. (2005). “Varieties of interpersonal rejection,” in *The Social Outcast: Ostracism, Social Exclusion, Rejection, and Bullying*, eds Williams K. D. Forgas J. P. Hippel W. von (New York, NY: Psychology Press) 35–51.

[B40] Levine J. M. Moreland R. L. (1994). “Group socialization: theory and research,” in *European Review of Social Psychology* Vol. 5 eds Stroebe W. Hewstone M. (New York, NY: John Wiley and Sons), 305–336.

[B41] Lev-Wiesel R. Sarid M. Sternberg R. (2013). Measuring social peer rejection during childhood: development and validation. *J. Aggress. Maltreat. Trauma* 22 482–492. 10.1080/10926771.2013.785456

[B42] Lincoln Y. S. Guba E. G. (1985). *Naturalistic Inquiry.* Beverly Hills, CA: Sage Publications, Inc.

[B43] Martín-Antón L. J. Monjas M. I. García Bacete F. J. Jiménez-Lagares I. (2016). Problematic social situations for peer-rejected students in the first year of elementary school. *Front. Psychol.* 7:1925 10.3389/fpsyg.2016.01925 PMC5156692 28018265

[B44] McDougall P. Hymel S. Vaillancourt T. Mercer L. (2001). “The consequences of childhood peer rejection,” in *Interpersonal Rejection*, ed. Leary M. R. (New York, NY: Oxford University Press), 213–247.

[B45] Mikami A. Y. Lerner M. D. Lun J. (2010). Social context influences on children’s rejection by their peers. *Child Dev. Perspect.* 4 123–130. 10.1111/j.1750-8606.2010.00130.x

[B46] Monjas M. I. Sureda I. García Bacete F. J. (2008). ?‘Por qué los niños y niñas se aceptan y se rechazan? [Why do children accept and reject each other?]. *Cult. Educ.* 20 479–492. 10.1174/113564008786542181

[B47] Mulvey K. L. Hitti A. Killen M. (2010). The development of stereotyping and exclusion. *Wires. Cogn. Sci.* 1 597–606. 10.1002/wcs.66 26271506

[B48] Mulvey K. L. Hitti A. Rutland A. Abrams D. Killen M. (2014). Context differences in children’s ingroup preferences. *Child Dev.* 50 1507–1519. 10.1037/a0035593 24491215

[B49] Nesdale D. (2007). “The development of ethnic prejudice in early childhood: theories and research,” in *Contemporary Perspectives on Social Learning in Early Childhood Education*, eds Saracho O. Spodek B. (Charlotte, NC: Information Age Publishing), 213–240.

[B50] Nesdale D. (2011). Social groups and children’s intergroup prejudice: just how influential are social group norms? *An. Psicol.* 27 600–610.

[B51] Nesdale D. Duffy A. (2011). Social identity, peer group rejection, and young children’s reactive, displaced, and proactive aggression. *Br. J. Dev. Psychol.* 29 823–841. 10.1111/j.2044-835X.2010.02012.x 21995740

[B52] Newcomb A. F. Bukowski W. M. Pattee L. (1993). Children’s peer relations: a meta-analytic review of popular, rejected, neglected, controversial, and average sociometric status. *Psychol. Bull.* 113 99–128. 10.1037/0033-2909.113.1.99 8426876

[B53] Nucci L. P. (2001). *Education in the Moral Domain.* Cambridge: Cambridge University Press 10.1017/CBO9780511605987

[B54] Orue I. Calvete E. (2011). Reciprocal relationships between sociometric indices of social status and aggressive behavior in children: gender differences. *J. Soc. Pers. Relat.* 28 963–982. 10.1177/0265407510397982

[B55] Rubin K. Bukowski W. Parker J. G. (2006). “Peer interactions, relationships, and groups,” in *Handbook of Child Psychology: Social, Emotional, and Personality Development*, Vol 3 eds Eisenberg N. Damon W. Lerner R. M. (Hoboken, NJ: John Wiley and Sons Inc), 571–645.

[B56] Saarento S. Kärnä A. Hodges E. V. E. Salmivalli C. (2013). Student–classroom–and school-level risk factors for victimization. *J. Sch. Psychol.* 51 421–434. 10.1016/j.jsp.2013.02.002 23816233

[B57] Sandstrom M. J. Zakriski A. L. (2004). “Understanding the experience of peer rejection,” in *Children’s Peer Relations: From Development to Intervention*, eds Kupersmidt J. B. Dodge K. A. (Washington, DC: American Psychological Association), 101–118. 10.1037/10653-006

[B58] Scandroglio B. López J. San José M. C. (2008). La teoría de la identidad social: una síntesis crítica de sus fundamentos, evidencias y controversias [social identity theory: a critical synthesis of its bases, evidence and controversies.]. *Psicothema* 20 80–89. 18206069

[B59] Selman R. (2003). *The Promotion of Social Awareness: Powerful Lesson from Partnership of Developmental Theory and Classroom Practice.* New York, NY: Russell Sage Foundation.

[B60] Smetana J. G. (2006). “Social-cognitive domain theory: consistencies and variations in children’s moral and social judgments,” in *Handbook of Moral Development*, eds Killen M. Smetana J. G. (Mahwah, NJ: Lawrence Erlbaum Associate), 119–154.

[B61] Smith G. D. (1950). Sociometric study of best-liked and least-liked children. *Elem. Sch. J.* 51 77–86. 10.1086/459206

[B62] Strauss A. L. Corbin J. (2007). *Basics of Qualitative Research*, 3rd Edn. Thousand Oaks, CA: Sage.

[B63] Suárez C. del Moral G. González M. T. (2013). Consejos prácticos para escribir un artículo cualitativo publicable en Psicología [Tips for writing a publishable qualitative article in Psycho]. *Psychosoc. Interv.* 22 71–79. 10.5093/in2013a9

[B64] Suárez C. Del Moral G. Martínez B. John B. Musitu G. (2016). El patrón de consumo de alcohol en adultos desde la perspectiva de los adolescentes [Adult pattern of alcohol use as perceived by adolescents]. *Gac. Sanit.* 30 11–17. 10.1016/j.gaceta.2015.06.004 26276406

[B65] Sureda I. García Bacete F. J. Monjas M. I (2009). Razones de niños y niñas de diez y once años para preferir o rechazar a sus iguales [Reasons why ten and eleven years old children prefer or reject their peers]. *Rev. Lat. Am. Psicol.* 41 305–321. 10.14349/rlp.v41i2.382

[B66] Thomas D. R. (2006). A general inductive approach for analyzing qualitative evaluation data. *Am. J. Eval.* 27 237–246. 10.1177/1098214005283748

[B67] Turiel E. (1998). Cultural practices, oppression, and morality. *Hum. Dev.* 41 166–171. 10.1159/000022576

[B68] Valles M. (2000). *Técnicas Cualitativas de Investigación Social.* Madrid: Síntesis.

[B69] Williams K. D. Zadro L. (2001). “Ostracism: on being ignored,” in *Interpersonal Rejection*, ed. Leary M. R. (New York, NY: Oxford University Press), 21–53.

